# Ignatian Spirituality as Inspiration for a Pedagogical Theory of Accompaniment

**DOI:** 10.1007/s10943-022-01628-z

**Published:** 2022-08-18

**Authors:** Zbigniew Marek, Anna Walulik

**Affiliations:** 1 Jesuit University Ignatianum in Krakow, Kopernika 26, 31-501 Kraków, Poland; 2 ul. Zaskale 1, 30-250 Kraków, Poland; 3 ul. Nazaretańska 1, 30-685 Kraków, Poland

**Keywords:** Spirituality, Ignatian spirituality, Health support, Ignatian pedagogy, Pedagogical accompaniment

## Abstract

The article presents Ignatian (Jesuit) spirituality as the source of a pedagogical concept that takes a subjective view of the human being regardless of their social position. Other subjects in the educational process (educator and personal God) are companions on the way to achieving good and developing one’s life potential. Another important aspect of Ignatian spirituality is the assumption that it can also perform a healing function, particularly transcendentally. This function concerns all the structures of human development and can be reflected in many ways of supporting human health. This perspective makes it possible to develop a model of Ignatian pedagogy in which the following elements are present: context, experience, reflection, action and evaluation. Recognising the educational values of these elements makes it possible to develop a pedagogical theory, called the pedagogy of accompaniment, which is reflected in a deep inner and outer sense of human existence. Thus, the purpose of this article is to describe the meaning of Ignatian spirituality for the pedagogy of accompaniment, with reference to its function of supporting the health of the human being, particularly the young.

## Introduction

A common definition of health, established by the World Health Organization (WHO) in 1948, specifies that health is ‘a state of complete physical, mental and social well-being and not merely the absence of disease or infirmity’ (Svendsen & Løber, 2020, p. 1). In 1984, WHO proposed a modified definition of health as "the extent to which an individual or group is able to realize aspirations and satisfy needs and to change or cope with the environment. Health is a resource for everyday life, not the objective of living; it is a positive concept, emphasizing social and personal resources, as well as physical capacities" (WHO. Regional Office for Europe, 1984).

Experience shows that a human life is influenced by both material and immaterial (spiritual) realities. These are both visible in health, which combines the material, mental and spiritual dimensions of an individual. In this sense, health is associated with integral human development. An individual having a coherent, integrated personality and positive relationships with other people finds it easier to solve everyday dilemmas. It conducive to their development and health, which is also revealed in the spiritual domain of their personality. However, to achieve such integrity, young people need the support of wise educators who are reflective, experienced practitioners in spiritual health as well as in other fields.

One proposal for helping young people reach maturity is educational accompaniment, which comprises the support in spiritual health that they receive. This concept takes many forms, but it seems that educational accompaniment—which draws on the 500-year-old Ignatian tradition (Lowney, 2003)—can be significant for contemporary education. The search for relationships between the material and spiritual realms was a trend in antiquity, when materiality and corporeality were placed at one pole and spirituality at the other.

The term *spirituality* is also found in the literature of the patristic era (fifth century). Originally, the term was given a religious significance, thereby exposing the inner experiences of the believer which arose from the relations that they had established with God and their social environment (Chmielewski, 2002, pp. 226–232). In this sense, spirituality refers to the progress or spiritual development of a person. The term was also often used to denote human religiosity.

In the social sciences, the question of spirituality is gaining an increasingly prominent place. It has shifted from religious reflection and been reinterpreted by relating spiritual content to spheres of human life such as awareness and self-awareness, wisdom, understanding, feelings, sensitivity, morality, creativity, aesthetic sense and world view. All these areas relate to health issues, both individually and socially.

### Developing Spirituality to Support the Health of the Human Being

We want to show Ignatian spirituality as the source of a pedagogical concept that supports the spiritual development of human beings. We assume that spirituality naturally supports health, which is understood as the well-being of the person. Moreover, referring to spirituality as it was originally understood—as religious spirituality—broadens the reflection on issues related to education and its relationship with health within education theory and practice. In Christian circles, the question of the importance of spirituality for the integrated development of a human being not only prompts reflection on the relations between these two realities, but also makes it possible to develop a specific pedagogy and even a pedagogical theory that would consider the positive spiritual influence on human health, which can be reflected in a person’s sense of well-being.

While spirituality was originally identified with or considered an aspect of religion, many currently describe it as existing outside of official church structures or even as an alternative to religion or religious traditions (Grom, 1992; Mariański, 2015). The term is sometimes associated with new religious movements. It is also sometimes identified with the human experience of that which is sacred and sometimes nameless, understood in terms of something personal, intimate and related to the power within a human being. The role of spiritual experiences and exercises is emphasised, as they cause the human body to be filled with an internal, spiritual meaning that manifests the creative forces of the Spirit (Mariański, 2015).

This role is often deprived of any theistic references, while the relationship between the person and their environment is highlighted. The WHO has referred to well-being in regard to such spheres of human life as awareness and self-awareness, wisdom, understanding, feelings, sensitivity, morality, creativity, aesthetic sense and world view (Socha, 2000). Such types of spirituality are called natural (or new) spirituality (Mariański, 2015; Marszałek, 2013).

Natural spirituality promotes respect for nature and healthy eating; it attaches great importance to interpersonal relationships based on fidelity and love; it emphasises an ethics that expresses respect for the moral values that it recognises; it is sensitive to art and stresses gender equality. This spirituality is holistic, democratic, accessible and non-hierarchical; it is placed in opposition to institutionalised religion—that is, it is beyond the church (Mariański, 2015, p. 39).

In the search for links between spirituality and health, it is also worth recalling the definition cited by Puchalski et al. (2014, p. 2). Although most often used in a clinical context, it defines spirituality as the aspect of humanity that refers to the way individuals seek and express meaning and purpose, and how they experience their connection with the present moment, with themselves, with others, with nature and with what is meaningful or sacred.

Different relationships between institutionalised religion and spirituality show how difficult it is to produce a unified definition of spirituality; however, whether natural or religious, the term refers to the category of health (Marek & Walulik, 2021) because by itself it is the subject of spontaneous manifestations of thoughts, speech, evaluations, aspirations, creativity and impulses that transcend worldliness and sensuality (Kunowski, 2011). It is also noteworthy that spirituality lends a distinct style to a person’s life, which arises from the attitudes they adopt, the habits (traditions) they cultivate and the values they recognise (Chmielewski, 2002; Fleming, 2008). It therefore reveals qualitative changes in human mental processes (i.e. thinking, perceiving and expressing emotions and evaluating what is good and what is bad).

At the same time, spirituality promotes a specific vision of life, a reflective attitude towards everyday life and a respectful attitude towards the world. Thus, health can be understood as a developmental potential. An echo of this style of thinking and acting is found in Ignatian spirituality. We will show how Ignatian spirituality can lead to the development of a pedagogical theory called the pedagogy of accompaniment (Marek, 2017). The centuries-old tradition of Jesuit education makes us aware that the assumptions of the pedagogy of accompaniment and their implementation in practice are conducive to the development of human potential, and thus health.

### Ignatian Spirituality

The ambiguity of the term *spirituality* stems from the historical and social conditions to which certain cultural traditions refer, as well as from the forms of life that are promoted by various social groups or institutionalised within particular denominations or intradenominational formational circles such as religious orders (Pawluczuk, 2004). One such form is Ignatian spirituality, inspired by Ignatius Loyola, the founder of the Jesuit Order (Society of Jesus). Fleming (2008) defines this type of spirituality as promoting attitudes, customs (traditions) and values that accompany the readiness to serve both God and humankind in the Church. This spirituality is characterised by the desire to devote oneself and one’s life completely to God by accompanying and cooperating with Jesus Christ in work related to the salvation of the world.

Ignatian spirituality uses spiritual exercises (retreats) to pursue human development. The aim is to order Ignatius Loyola’s vision of man and connect it with the supernatural world. It is about the relationship between nature and grace, the Creator and the creature, the Saviour and the saved world (An Outline of the Spiritual Exercises, 2022). Loyola communicated this vision in the Constitutions of the Society of Jesus (Hajduk & Mółka, 2007; Inglot, 2007) and in other writings, chief among them being the very practical booklet *Spiritual Exercises*.

Many editions of the book have been published. Loyola used the term spiritual exercises to refer to the examination of one’s conscience, meditation, contemplation, oral and mental prayer and all other spiritual activities (Loyola, 2018). The content of this book is meant to help a person conquer themselves and regulate their life without succumbing to any disordered tendencies (Królikowski, 2010; Poznański, 2010), which—when paired with an integral understanding of the person—almost directly relates the expected effects of exercise to the category of health.

### St Ignatius’ *Spiritual Exercises* as the Way to Maturity

These spiritual exercises themselves are considered a method for developing the individual or a kind of pedagogy that supports individuals in undertaking integral work on their character. The form of spirituality proposed by Ignatius Loyola helps a person to order their inner and outer life; that is, it fosters self-education (Augustyn, 2010; Królikowski, 2010; Wójtowicz, 2010), which takes the form of theocentric human formation (the concepts of education and formation will be used interchangeably to emphasise that education is an essential component of formation). It sensitises a person to the need to acquire the skills of conquering oneself and ordering one’s own life (Horowski, 2020) and to thereby acquire the virtue of ‘self-management’ (Gurba et al., 2017) that opens one up to God and other people. Ignatian spirituality aims to help a person reach maturity by shaping their world view, vision for their life and ideals according to self-awareness, creativity, love and heroism (*The Characteristics of Jesuit Education*, 1986).

Such an attitude results from Ignatius’ belief that God is the Creator and Lord, the Supreme Good and the only absolute Reality and that humans are both of God and for God, while God is the goodness and happiness in human life (Mullen, 2018). This means that in the course of a person’s formation, they should discover the mystery of a personal God who is both the source and giver of all that is good (Marek & Walulik, 2020) and who establishes personal relationships with people (Lowney, 2003). In this context, Ignatius asserts that God is not at the service of humankind but, conversely, that people should be engaged in service to God (Ganss, 1991). The motive for promoting such an attitude should be one’s inner faith and the conviction that God reveals Himself to people as a Light that dispels the darkness caused by evil and that a person on their own will not be able to free themselves from it.

### Context, Experience, Reflection, Action and Evaluation in the Formation of the Person

Another characteristic feature of the model of formation that grows out of Ignatian spirituality is the context of retreatants’ lives. The retreat director should learn about the retreatants’ attitudes towards God and prayer because, in Ignatius’ view, only openness to the action of the Holy Spirit may allow a retreatant to change their own life. Another important step in this method is gathering the experiences of the retreatant. Ignatius meant to allow the retreatant to feel internally with their mind, heart and will that God the Creator shares Himself and His life with His creation (Królikowski, 2010).

Another element in the formation of a retreatant is reflecting on the meaning of these experiences. Ignatius defined this action as ‘discernment’. It is intended to help the retreatant become aware of the inner motivations and reasons that underlie their judgements or decisions and to evaluate them in light of the consequences that might arise from striving for a particular goal (Arrupe, 1967; Sheets, 1969). The result of this discernment should be the generosity of the retreatant, as their decision in response to God’s love is now known to Him. For Ignatius, this meant a readiness to act in accordance with God’s will in one’s life, give Him due worship, cooperate with Him and follow the example of Jesus Christ for our own salvation and the salvation of the whole world.

The fruits of such reflection are specific decisions to be made (the choice of specific values). The last element in Ignatius Loyola’s model of spiritual formation is evaluating one’s achievements thus far. It is characteristic that the retreatant is the one who evaluates; Ignatius encourages them to examine themselves in order to become aware of their victories and failures in meditation or contemplation (Królikowski, 2010; Loyola, 2018). Both victories and failures can be helpful in integrating the retreatant, whose sense of well-being may be associated with the sense of good health, understood as both natural and spiritual health.

### Spiritual Director

Another feature of human formation based on Ignatian spirituality is the role of the retreat director throughout the process. Ignatius became convinced of the need for a spiritual director during his stay in Manresa. The internal anxieties, dilemmas and doubts that Ignatius himself experienced during this period convinced him that various kinds of feelings are intensified in the soul of the retreatant, as are the related internal states of consolation, desolation or anxiety.

All this confirmed Ignatius’ belief that retreatants need help from someone who is familiar with their experiences: a retreat director. Their task is to select and adapt individual exercises to the internal state of the retreatant. They can share their own experiences with the retreatant, i.e. give them a testimony of their own experiences and the decisions that they have taken (Marek & Walulik, 2019). However, the retreat director must be restrained and must avoid pressurising the retreatant, so that they can take decisions and make choices that are significant for themselves, both independently and freely (Jalics, 2016).

As stipulated in the ‘Presupposition’ (the part of the instructions intended for the retreat director), a retreat director should be benevolent and willing to give the retreatant the benefit of the doubt (Gałkowski & Kaźmierczak, 2021). The inner order to which the successive stages of spiritual exercises lead, under the supervision of the director, is an indispensable factor in both health (whether it is well-being according to the WHO definition or potential) and education.

### Ignatian Pedagogy

The cooperation between a retreatant and a retreat director arising out of Ignatian spirituality and used in spiritual exercises has become the prototypical model of pedagogy for the Jesuits in their school education; it is called Jesuit (Ignatian) pedagogy. It should be noted here that Ignatius Loyola did not originally plan for the order he founded to be involved in education; however, quite quickly, he and other Jesuits understood the paramount role that schools play in forming a mature person (Inglot, 2007).

During Ignatius Loyola’s lifetime, the schools opened by the Jesuits already integrated the humanistic and Christian dimensions of human life; thus, they sought to combine anthropology and theology. This direction of education was not an obvious choice, if only because Ignatius Loyola and his contemporary Jesuits were culturally people of the Middle Ages, which made it difficult for them to understand and accept the emerging concept of humanism, as noted by Piechnik (1990).

However, this fact did not prevent them from opting for an innovative (humanistic) model of education. Ignatius Loyola borrowed this model from the Spanish educator Juan Luis Vives. He opted to use the Spanish curriculum, which was likely also chosen in order to follow the humanistic model.

On the other hand, when selecting the system and organisation of education, he followed the example of the University of Paris. Loyola valued this system primarily because of its didactic and educational method, whereby students were divided according to their age and knowledge and were required to attend scheduled lectures and to pass exams. In other universities, there was freedom in this respect. Ignatius appreciated the fact that the Parisian professors encouraged their audiences to discuss, make independent statements about and care for their religious education.

### Fundamentals of the Jesuit Education System

Over the following years, the Jesuits developed their own education system, using the educational models mentioned above. Apart from teaching the humanities, their system included lectures in logic, philosophy of law, natural philosophy, moral philosophy, metaphysics, scholastic and positive theology and the Holy Bible. In addition, they taught history, modern languages and mathematics applied to military art and civil architecture. The school curricula also included military exercises with weapons, horse riding, dancing, drawing, etc. (Piechnik, 1990).

The education system was developed so as to provide students with a thorough intellectual background and to develop in them critical, emotional, moral and spiritual sensibilities in order to make them more mature and responsible for their own lives (Marek, 2017; Mółka & Mółka, 2019; Piechnik, 2003; Puchowski, 2007; Żmudziński, 1995). It is not without significance that the Jesuits emphasised the development of each student—both those more and less able—not only physically and intellectually, but also psychologically and spiritually.

The terms ‘*magis*’ and ‘*cura personalis*’ became the signature strengths of the Jesuit philosophy of education. Both terms characterise the relationship between the teacher/educator and the student/pupil, as well as educational plans and other aspects of the life of educational institutions. Both terms require Jesuit schools to care for each student and thus for their multidimensional development (Piechnik, 1990, 2003; Puchowski, 2007; Żmudziński, 1995).

The first of these terms is drawn directly from *Spiritual Exercises* and from the retreatants’ discovery that they were created to serve God in full reverence. The discovery of this truth is supposed to inspire love, which in its essence is limitless and always wants more and causes one to strive for that which is highest. Love understood in this way is expressed by the word *magis*, i.e. more or better. Ignatius meant that we should always choose and desire solutions in life that will contribute to a more thorough achievement of our goals (Ganss, 1991).

The Jesuits also transferred expectations arising from the principle of *magis* to education, and they complemented this principle with the notion of *cura personalis*. In the Jesuit philosophy of education, this term means care, concern, attention and even the teacher’s love for each student, as well as trust between the two parties. Although the concept is rooted in Ignatian spirituality, the term itself was first used in 1934 by the then Superior General of the Society of Jesus, Włodzimierz Ledóchowski. He used the term in a speech given to the representatives of high schools and universities run by Jesuits in the USA (Casalini, 2019).

### From Educational Projects to the Jesuit Model of Education

The Jesuits’ work on their own model of education began to yield results in the form of written projects, the first of which was announced in 1558. Over the years, it was subject to revision and corrections, and the final version was published in 1599 under the title *Ratio atque Institutio Studiorum*. This document was in force until the dissolution of the order in 1773. It describes the methods of achieving specific and practical educational goals in Jesuit schools (Inglot, 2007; Piechnik, 2003).

**Fig. 1:**
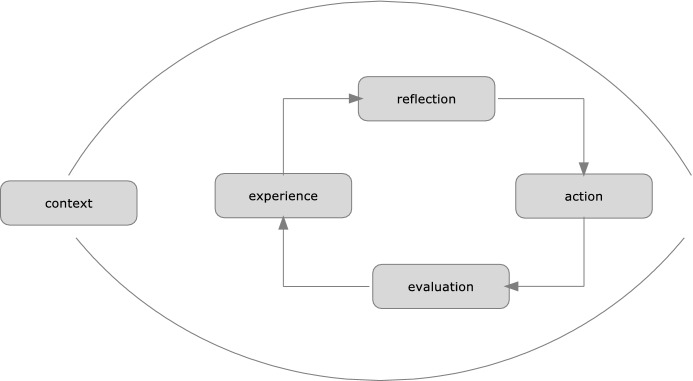
Diagrammatic interpretation of Ignatian education (based on Society of Jesus (1993))

The need to reconceptualise this instruction according to the vision of a person promoted by Ignatius Loyola and to adapt it to contemporary educational needs was verbalised in the second half of the twentieth century. Several years of work resulted in the publication of two documents, ‘The characteristics of Jesuit education’ and ‘Ignatian pedagogy: A practical approach’. These papers describe the model of Jesuit education that grew out of Ignatian spirituality. They emphasise that integral development requires people to be open to both the horizontal and vertical dimensions, the latter being theocentric (Bitter, 2003; Codina, 2007). According to these publications, the Ignatian model of education is never directed against the person and their true good (Augustyn, 2010; Mateo, 2008). This is illustrated by the diagram in Fig. 1, which presents the essential elements of the spiritual exercises proposed by Ignatius Loyola.

### Context, Experience, Reflection, Action and Evaluation in the Jesuit Model of Education

In updating the model of education, careful attention was paid to the importance of sociocultural/religious factors in a student’s education. It was pointed out that the teacher/educator needs to understand the student’s previous experiences, because education does not take place in an axiological void, but rather under certain circumstances that determine its course. With this context in mind, it was postulated that the teacher should try to become familiar with the student’s world, which is in fact a point of reference for both teaching and learning.

This postulate encompasses getting to know a student’s socio-economic, political and social conditions, as well as their expectations, beliefs, predispositions, achievements or shortcomings. This context is understood to comprise the student’s views and way of thinking, as well as their feelings, attitudes and values. Thus, the teacher’s knowledge of these contexts can help them establish a personal relationship with the student. The context of the ongoing educational process within Ignatian pedagogy creates space for a fuller understanding of the individual elements of the whole educational model.

The first component of the educational process is the reference to both natural and religious experience. By experience, we understand individual events in the life of a person which leave a permanent mark. Each type of experience covers both the sensual and the spiritual aspects, both external and internal reality. It is in the nature of any experience that it cannot be forced, but only felt and accumulated.

Each experience arouses interest in a person and prompts them to ask questions and express opinions. Each experience triggers hope in regard to transforming one’s own life and stimulates various anxieties, which has a healing effect on the integrated human being. Although concrete experiences belong to only one person, other people may have similar—though not identical—experiences (Marek, 2017).

According to the assumptions of Ignatian pedagogy, the aim of recalling experiences is to trigger a pupil’s activity by engaging their cognitive and emotional spheres. This leads to the second stage of the process, which is called reflection and whose purpose is to identify the internal motives and reasons that prompt a person to make certain judgements and to examine the causes and implications of their own experiences. It can be described as extracting the meanings of accumulated experiences, of a given topic, of an idea or task or of a spontaneous reaction that shows the meaning of everything in which the person participates (Chrost, 2021). The advantage of this activity is that it fosters the student’s understanding of why they have adopted certain positions or attitudes. Reflection is also about considering the possibility of making choices while being aware of their likely consequences.

Finally, reflection refers to a pupil’s discovery of what leads them to the desired level of freedom. Thus, it follows that reflection brings out the meaning of the human experience and fosters the ability to take responsible decisions (Brzostek & Walulik, 2020; Szymańska, 2018). It is helpful for the process of reflection if we are able to use the two available sources of knowledge: natural, which is produced by the intellect and the senses, and supernatural, the truth of which is discovered in God’s revelation (Marek, 2014).

The next component in the model of Ignatian pedagogy is action. The focus on action is meant to prepare students to make intelligent and effective contributions to their communities, and it emphasises that reflection is insufficient and incomplete if it does not lead to concrete action. A student’s readiness to act is crucial in taking life decisions that are inspired by the attitudes, values and ideals adopted in accordance with the Ignatian principle of *magis* (Marek, 2017). Nadal (1962) called St Ignatius *in actione contemplativus* [contemplative in action]—a man living in union with the Lord Jesus, according to the principle that ‘in Him [Jesus] we live, move and exist’ (*Good News*, 1997, Acts 17:28).

Others refer to this lifestyle as walking in the presence of God or finding God in all things (Coreth, 1954; García-Villoslada, 1990; Grogan, 2021; Martin, 2010). The choice itself should be a gradual identification of the pupil’s priorities that arise from the truth they have discovered. The competences acquired in this approach may be conducive to making an adequate evaluation and self-evaluation (Brzostek & Walulik, 2020) that is conditioned by the student’s age, abilities and level of development.

The evaluation stage is the final component in the model of Ignatian pedagogy, in which a pupil takes a critical look at their previous achievements in the cognitive, emotional and behavioural spheres. It should be an assessment of one’s internal growth (Kotlewski, 2005) in light of an ideal, not of perfection in itself or the successes that one has accomplished through unhealthy competition or excessive self-absorption. In other words, evaluation helps the student discover and understand what it means to be a human being (Marek, 2017) and what should be done to improve the current state of one’s existence in order to be ‘healed’ from any weakness that would disturb further developmental progress. Taking into account this argument and the fact that the school is committed to the integral development of a person who should become ‘a person for others’, the Jesuits emphasise the importance of a pupil periodically evaluating their own progress in regard to their attitudes and chosen priorities (Marek & Walulik, 2021).

### The Teacher/Educator in the Jesuit Model of Education

In the educational model proposed by the Jesuits—in addition to the challenges faced by the pupil/student—an important role is assigned to the educator/teacher, similar to that of the retreat director in spiritual exercises. This role cannot be reduced to instruction or indoctrination, as these methods suppress the spirit; nor can it be reduced to suggesting certain behaviours. The crucial aspect of the teachers’ role is bearing witness, i.e. sharing their own view of life as seen from a human and divine perspective. Through witnessing and sharing, the tutor helps the pupil understand what true inquiry is and what solving important existential issues or choosing values consists of.

For the pupil, such accompaniment relates to acquiring knowledge, preparing for a profession and attaining the ability to live harmoniously in the community, i.e. becoming a mature person. This in turn shows the pupil that accompanying is not a project, plan or idea. Instead, it consists of lending credibility to the knowledge, values and attitudes conveyed to the pupil in order to help them feel what they think and do, help them do what they think and feel and help them use the language of the head, heart and hands simultaneously (Schwietert Collazo & Rogak, 2013).

An example of such an accompaniment is the Jesuit theatre (Puchowski, 2007), whose influence on both the actors and the audience is meant to stimulate greater sensitivity to the good. It is supposed to teach what it means to be a man or woman for others (Duminuco, 2008) in the spirit of *magis* and *cura personalis* (Mółka & Steczek, 2008). Following these principles fosters the intellectual, emotional, moral and spiritual development of a student; it also awakens in them a sense of their own dignity and value and finally makes them aware that they are participating in the life of a larger community for which they are responsible (Marek & Walulik, 2019). This also helps them overcome developmental obstacles and obtain a better state of well-being through the healing function of professionally conducted spirituality.

### The Pupil in the Jesuit Model of Upbringing

A pupil’s development in the course of their education depends on how well they understand that they are the subject of the educational process. This problem has been highlighted in studies on the ‘coexistence’ and ‘co-creation’ of research subjects, i.e. student and teacher. It has been noted that the researcher meeting with the subject is a sine qua non condition for the effectiveness of the educational process. It has been shown that the researcher establishes a dialogue not only with the research subject, but also with themselves (Patton, 1990).

Collaboration and cooperation (including the research subjects throughout the research), as well as co-creation (mutual interaction between two full-fledged subjects of research), indicate the formative dimension of the relationships established between these subjects (Ciechowska, 2020). Thus, these relationships make us aware that the tasks Ignatian pedagogy assigns to an educator can be fulfilled when there is a relationship between the subjects of the educational process (the pupil and the educator) that expresses mutual respect and trust, thereby resulting in a change in the established patterns of thinking in both subjects.

The integral understanding of the person, which is the basis for achieving inner coherence through Ignatian spiritual exercises in educational practice, is expressed by building positive relationships with other people. This is done by developing the physical, mental, social and spiritual potential of the person—the same areas that determine human health. This reflection prompted the development of the theoretical assumptions of educational activities.

### From Ignatian Spirituality to the Pedagogy of Accompaniment

Pedagogy inspired by Ignatian spirituality can be used to build general propositions and form the foundations of the pedagogical theory called the pedagogy of accompaniment, i.e. Jesuit pedagogy (Marek, 2017). This can be compared to a cognitive/practical intellectual map created by cognition, research, description and explanation, as well as the interpretation of a person participating in the educational process (Tchorzewski, 2016). The theory itself is based on the philosophical and theological assumptions of Christian personalism, which views humankind as persons, the unity of body and soul. This person came from God and, as a free agent, aims to return to Him, thereby transcending their own limitations. Christian personalism also emphasises that a human being is not a lonely island, but instead lives in a social community within a specific culture (Mółka, 2016).

### The Pedagogy of Accompaniment Within the Social Sciences

Usually, the pedagogy of accompaniment is situated on the border between pedagogy and theology (Hilger et al., 2001; Kunstmann, 2010; Mette, 1994). Therefore, it is a discipline of contemporary humanities and modern theology that uses a hermeneutic perspective to describe the educational process (Marek, 2016; Milerski, 2011). The extant research on the subject uses methods that are typical of both the social sciences and theology (Milerski, 2020). The subject of these disciplines is the development of humankind seen from the natural and the supernatural (religious) dimensions. This perspective can shed light on the internal processes that take place in a person as a result of the relationships established both with others and with their personal God.

### The Research Subject and Functions of Accompaniment Pedagogy

As a pedagogical theory the pedagogy of accompaniment deals with cognition, studying, describing, explaining and interpreting a fragment of social reality, which is the process of educational accompaniment. As it stems from the Ignatian tradition, the source of knowledge of this process is experiences interpreted through the spiritual exercises of Ignatius of Loyola. In order to be studied, they must be viewed through the spirit of the humanistic paradigm (e.g. subject participatory or synergistic/participatory) using qualitative methods of data collection, analysis and interpretation. Similarly, spiritual exercises and Ignatian pedagogy appreciate the subjectivity of the educator and the pupil, while the research related to the pedagogy of accompaniment appreciates the subjectivity of the researcher and the subject.

This theory of educational accompaniment performs the functions attributed to pedagogical theories, namely diagnostic, evaluative and prognostic. The diagnostic function of the pedagogy of accompaniment is realised by valuing the contexts in which an experience takes place. The contextual interpretation of the experience makes it possible to diagnose the life situation of both the pupil and the educator.

According to the assumptions of the pedagogy of accompaniment experience should be reflected upon, allowing one to identify the causes of a given situation and to form a plan for achieving a particular goal. In performing the evaluative function, the pedagogy of accompaniment verifies that the actual state of experience corresponds with a certain ideal; it uses the achievements of transcendent pedagogy, which provides access to knowledge through natural and religious knowledge.

The primary advantage of this approach is that it provides new knowledge and enables a person to broaden their horizons regarding the key questions about human existence. The prognostic function requires hypotheses about the future of the pupil. The pedagogy of accompaniment derives these hypotheses from analysed experiences, thereby basing the research process on respect for the dignity and freedom of the individual.

This theory also has a therapeutic function, reflected in the continual process of healing from the many difficulties and hardships that are faced along the way. Understanding them from the proper perspective becomes a benefit derived from the mature accompaniment of an educator whose spiritual competences are very high. Such an educator is likely to share their experience from the spiritual/healing path that they have taken.

### The Purpose of Education in the Pedagogy of Accompaniment

The aim the pedagogy of accompaniment is to open up the possibility of referring to transcendence in the course of education—i.e. to God, who is a subject of the process, along with the pupil and the educator. Invoking the personal relationship between an individual and God helps us explain everyday life in transcendent categories and to draw pedagogical consequences from them. The purpose of the pedagogy of accompaniment is not to alienate people from reality, but rather to inspire them to discover it from a religious (Christian) perspective.

This is evidenced by the fact that the aims of the pedagogy of accompaniment are divided into two levels: the immediate aims and the final goal of life. The first-level aims encompass acquiring the knowledge, skills and attitudes that are indispensable to everyday life, whereas the final goal in the pedagogy of accompaniment is connected with the relationships that people establish and develop with their personal God, who invites them to live with Him for all eternity (Marek, 2017).

As the pedagogy of accompaniment defines the goal of human life and advocates striving for maturity, a student is taught to use not only natural, empirical and intellectual knowledge, but also supernatural knowledge derived from divine revelation (Marek, 2014), which is based on the authority of the personal God. Invoking the authority of God gives the pedagogy of accompaniment a formative dimension, which provides the pupil’s life with a specific style. It also enables a person to transcend their own limitations and introduces them to a new reality that is discovered through supernatural (religious) knowledge, which becomes a new quality in the process of transforming one’s current state into a better one. The fact that this reality is not accepted by everyone does not mean that it does not exist.

### Ways to Achieve the Goals Proposed by the Pedagogy of Accompaniment

To achieve its goals, the pedagogy of accompaniment uses proven patterns of action, in which the accumulated experience, the reflection on such experience and the resulting action all play a special role. It is assumed that the activities which support a person in acquiring deeper knowledge—e.g. in research or describing, explaining and interpreting reality—have an impact on the educational process (Tchorzewski, 2016) and thus on the person’s attainment of maturity or perfection.

Finally, it should be noted that the method of examining the educational reality that is developed through the pedagogy of accompaniment does not claim to be a coherent system of statements concerning the entire educational reality, and thus does not claim the exclusive right to describe it. The pedagogy of accompaniment in the Ignatian tradition is part of the idea of lifelong learning, especially through communities of practice and learning by experience. This kind of educational activity can also become a way of collecting empirical data in qualitative pedagogical research, such as action research (O’Grady, 2010).

## Final Conclusions

The aim of the argumentation presented herein has been to investigate how a specific spirituality can lead to the development of a pedagogical theory. Moreover, this argumentation justifies the purpose of the article, that is, to explore the meaning of Ignatian spirituality for the pedagogy of accompaniment, with reference to its function of supporting the health of the human being, particularly the young. The benefits of the pedagogy of accompaniment also include the healing function, which has a special therapeutic function perceived in the spiritual dimension.

All in all, it is not surprising that Ignatian spirituality was chosen as an example of spirituality, as it seeks to give human life a specific style characterised by a combination of theocentrism and anthropocentrism. It turns out that this particular type of spirituality has been an inspiration for a specific educational enterprise—Ignatian pedagogy—which is characterised by a specific approach to the educational process, namely emphasising the roles of both the pupil and the educator. The educational process is taken to be their common path, although each has a different task to fulfil.

Thus, the pupil, by discovering and reflecting on the experiences that they have accumulated, is supposed to make life decisions both independently and freely. The educator, on the other hand, should be a fellow traveller in this journey, a witness explaining to the pupil their states, doubts and enquiries. They should avoid pressurising or interfering with the decisions of the pupil. They may, however, inspire the pupil to evaluate the significance and consequences of the decisions that they take.

The pedagogy that grew out of Ignatian spirituality has enabled us to lay the foundations of a pedagogical theory, which we have named the pedagogy of accompaniment. This pedagogy of accompaniment is characterised by the presence of three subjects in education. The first is the pupil themselves, endowed with and making use of freedom. The other two subjects are the educator and the personal God.

The relationships developed between these subjects favour the development of the type of person whom the pedagogy of religion describes (Marek & Walulik, 2020). These observations lead to the conclusion that Ignatian spirituality is conducive to the development of the pedagogy of accompaniment. Referring to both the natural and supernatural (religious) reality, this theory promotes and teaches respect for the values discovered through both natural and supernatural cognition.

By its nature, this pedagogy is located within the Christian strand of religious pedagogy. Educational activities carried out according to the pedagogy of accompaniment authorise one to understand the category of health, as it relates to not only well-being but also developmental potential. Thus, the appeal of the educator and pupil to particular dimensions of health, including the spiritual and religious dimensions, promotes the quantitative and qualitative release of the individual’s potential.

## Limitations

What this study offers is only a theoretical model. It still requires empirical research to provide verification. Therefore, further studies are called for in order to yield a more grounded and sound theory that would be better embedded in practice.

## Data Availability

Not applicable.
